# *VaAPRT3* Gene is Associated With Sex Determination in *Vitis amurensis*

**DOI:** 10.3389/fgene.2021.727260

**Published:** 2021-12-23

**Authors:** Yan Men, Ji-Rui Li, Hai-Lin Shen, Yi-Ming Yang, Shu-Tian Fan, Kun Li, Yin-Shan Guo, Hong Lin, Zhen-Dong Liu, Xiu-Wu Guo

**Affiliations:** ^1^ College of Horticulture, Shenyang Agricultural University, Shenyang, China; ^2^ Institute of Pomology, Jilin Academy of Agricultural Science, Gongzhuling, China; ^3^ Institute of Special Wild Economic Animal and Plant Science, Chinese Academy of Agricultural Sciences, Changchun, China

**Keywords:** sex, VaAPRT3, marker, cytokinin, *Vitis amurensis*

## Abstract

In the past decade, progress has been made in sex determination mechanism in *Vitis*. However, genes responsible for sexual differentiation and its mechanism in *V. amurensis* remain unknown. Here, we identify a sex determination candidate gene coding adenine phosphoribosyl transferase 3 (*VaAPRT3*) in *V. amurensis*. Cloning and sequencing of the *VaAPRT3* gene allowed us to develop a molecular marker able to discriminate female individuals from males or hermaphrodites based on a 22-bp InDel. Gene expression and endogenous cytokinin content analysis revealed that the *VaAPRT3* gene is involved in sex determination or, to be precise, in female organ differentiation, through regulating cytokinin metabolism in *V. amurensis*. This study enlarged the understanding of sex determination mechanism in the genus *Vitis*, and the sex marker could be used as a helpful tool for sexual identification in breeding programs as well as in investi*g*ation and collection of *V. amurensis* germplasms.

## Introduction

Grapevine (*Vitis* L.) is an important temperate fruit crop widely used for fresh fruit supply, raisin production, and juice and wine making. In China, the area for grapevine cultivation was nearly 0.75 million ha with a harvest of 14.37 million tons (FAO, 2019). China is also abundant with wild grapevine resources. The number of *Vitis* species that originate in China accounts for approximately 50% of the world total (Wan et al., 2008). Among these species, *V. amurensis* has the strongest cold hardiness and is resistant to various fungal diseases. It has been an important material for sweet red wine making in China, and has also been the most important germplasm resource in grapevine cold hardiness breeding.

Similar to *V. vinifera* subspecies *sylvestris*, *V. amurensis* is mostly dioecious. The female plants produce flowers with perfect formed pistil having style and stigma but reflexed stamens with infertile pollen, but the male plants have erect stamens and fertile pollen, but aborted pistils with no style or stigma (Caporali et al., 2003; Wang et al., 2013; Shen et al., 2018). Based on the model proposed by Antcliff (1980), the inheritance of sex in grapevine is thought to be controlled by a single major locus with three different alleles, male (M), female (F), and hermaphrodite (H) in the following allelic dominance M > H > F. Thus, plants carrying the male allele produce male flowers and only plants homozygous for the female allele exhibit female phenotype. This theory has been proven in not only European grapevines (Marguerit et al., 2009; Fechter et al., 2012; Battilana et al., 2013), but also American (Lowe and Walker, 2006; Riaz et al., 2006) and Asian species (Wan et al., 2001), including *V. amurensis* (Yin et al., 1997).

Grapevine sex locus has already been identified in several genetic mapping studies, and some candidate genes with potential impact on sex determination were also predicted. Dalbó et al. (2000) located the sex locus near the Single Sequence Repeat (SSR) marker VVS3 on linkage group 14, and that matches chromosome 2 when compared with the subsequent *V. vinifera*-derived reference genetic linkage maps (Adam-blondon et al., 2004; Riaz et al., 2004). Lowe and Walker (2006) and Riaz et al. (2006) located the locus near another SSR marker VVIB23 by using mapping populations of different genetic backgrounds. Marguerit et al. (2009) confirmed the position of sex locus on linkage group 2, close to VVIB23, and considered the gene encoding the enzyme 1-aminocyclopropane-1-carboxylic acid synthase (*ACS*) as a putative gene for the control of sexual traits in grapevine. Fechter et al. (2012) identified a candidate region of 143 kb located between 4,907,434 and 5,050,616 bp of chromosome 2 on the *V. vinifera* physical map, and developed a marker able to discriminate female plants from hermaphroditic/male ones by analyzing a candidate gene *APRT* in detail. Picq et al. (2014) enlarged the sex region to 154.8 kb and found several transcripts with significant polymorphism between male and female genotypes. Hyma et al. (2015) located the sex locus to a physical position between 4.75 and 5.39 Mb of the *V. vinifera* physical map with three populations, further supporting the previously identified location of grapevine flower sex (Fechter et al., 2012; Battilana et al., 2013; Picq et al., 2014). Coito et al. (2017) found two genes that together allowed the distinction among male, female, and hermaphroditic *V. vinifera* plants. Based on the two genes identified by Picq et al. (2014), Conner et al. (2017) developed markers for sex in muscadine grape (*Vitis rotundifolia*). Recently, Lewter et al. (2019) mapped the muscadine grape sex locus to an interval that aligned to 4.64–5.09 Mb on chromosome 2 of *V. vinifera* physical map, which included the 143 kb region defined by Fechter et al. (2012). Massonnet et al. (2020) characterized the grapevine sex-determining region and developed a sex marker based on an 8-bp InDel within a novel male-fertility candidate gene *INP1*. Based on the above studies, several genes might be involved in sex determination in grapevines. Among these genes, the *APRT3* gene may play an important role. Firstly, *APRT* gene has been successfully used in development of sex markers in *V. vinifera* (Coito et al., 2017) as well as in more complex *Vitis* genetic background (Fechter et al., 2012). Secondly, *APRT* gene was reported to have a potential role to catalyze cytokinin conversion from free bases to nucleotides (Allen et al., 2002; Zhang et al., 2013), and this process may affect floral organ development in plants (Moffatt and Somerville, 1988; Regan and Moffatt, 1990; Gaillard et al., 1998; Zhang et al., 2002; Zhou et al., 2006). The importance of cytokinin in sex formation has also been shown in grapevine by converting male flowers to hermaphroditic ones when applying exogenous cytokinins (Negi and Olmo, 1966; Zhu, 2017; Shen et al., 2018). What is more, sex determination was thought to be influenced by the different expression level or dosage of certain alleles of *APRT* gene in grapevine (Massonnet et al., 2020).

Traditional mapping of important traits based on genetic linkage map construction requires genotyping of large segregation populations, and is time-consuming and labor-intensive. However, Michelmore et al. (1991) developed a bulked segregant analysis (BSA) method to rapidly detect markers linked to the trait of interest. When combined with the next-generation sequencing (NGS) technology, the BSA method can be used as a fast track approach to locate candidate genomic regions more rapidly (Singh et al., 2016). The approach of BSA + NGS has been successfully used in qualitative trait gene mapping (Teng et al., 2017) and also in analyzing quantitative trait that exhibits a pair of extremely contrasting phenotypes (Takagi et al., 2013; Chen et al., 2017; Liu et al., 2017; Xue et al., 2017; Yin et al., 2018).

The aim of this study was to fine map the sex locus and to identify candidate gene with potential impact on sex determination in *V. amurensis*. To this end, we combined the NGS-based BSA and traditional linkage analysis to construct a linkage group containing the sex locus. Based on cloning and sequencing of a candidate gene *VaAPRT3*, a molecular marker able to discriminate female individuals from males or hermaphrodites was developed, and the universality of the marker was tested across different *V. amurensis* germplasms as well as an additional cross population. To explore the role of the *VaAPRT3* gene in sex determination in *V. amurensis*, we also analyzed the relationship between its expression pattern and the contents of endogenous cytokinins in different floral developmental stages of three sexes. The results of this study help enlarge the understanding of sex determinism of the genus *Vitis*, and provide a helpful tool for sexual identification in both breeding programs and investigation and collection of *V. amurensis* germplasms.

## Materials and Methods

### Plant Material, Phenotyping of Sex, and Flower Sampling

Four pure *V. amurensis* materials, “Zuoshanyi”, “043”, “75042”, and “Shuangqing”, were used as parents to generate cross populations. “Zuoshanyi” is a female cultivar released in China in the year 1984 (Song et al., 1999). “043” and “75042” are both male germplasms. “Shuangqing” is the only natural hermaphroditic germplasm discovered in northeast China and was registered as a cultivar in 1975 (Lin, 1982).

An F1 population (named “Z0”) of 117 individuals was generated by crossing “Zuoshanyi” and “043”, and was planted at the Institute of Pomology, Jilin Academy of Agricultural Science (Gongzhuling City, Jilin Province, China; 43°31′N, 124°49′E). Another two cross populations were obtained by the cross “Zuoshanyi” × “75042” (named “Z7”) and “Zuoshanyi”× “Shuangqing” (named “ZS”), and they are both growing in the vineyard of Shenyang Agricultural University (Shenyang City, Liaoning Province, China; 41°48′N, 123°25′E).

Each of the three populations segregates for sex (female vs. male for “Z0” and “Z7”, female vs. hermaphroditic for “ZS”). The common female parent “Zuoshanyi” possesses two female alleles, and each of the two male parents carries both a male and a female allele and the hermaphroditic parent “Shuangqing” carries both a hermaphroditic and a female allele. Individuals of the populations were scored for sex during the flowering period by visual inspection using the “Descriptors and data standard for grape (*Vitis* L.)” (Liu et al., 2006). For all the three crossing populations, the goodness of fit of the actual segregation ratio to the theoretical expected ratio was tested by the *χ* ^2^ test.

The “Z0” population was used for linkage group construction, and the other two populations consisting of 126 and 93 individuals, respectively, were used for marker verification. A small group of dioecious *V. amurensis* germplasms distributed in different areas of the Northeast three provinces in China was also used for marker verification, and they were collected in the 1970s and 1980s and now conserved in the National Field Gene Bank for Amur Grapevine (Zuojia Town, Jilin Province, China; 43°57′N, 125°59′E). Information of all the germplasms used in this study is shown in Supplementary Table S1.

### DNA Extraction and Bulk Construction

Young leaves were collected and immediately frozen in liquid nitrogen and stored at −80°C until DNA extraction. The genomic DNA was extracted using an improved cetyl trimethylammonium bromide (CTAB) method (Hanania et al., 2004). DNA was quantified with a NanoDrop 2000 spectrophotometer (NanoDrop, Wilmington, DE, United States). Two DNA bulks, a female bulk (F-bulk) and a male bulk (M-bulk), were prepared by mixing equal amounts of DNAs from 30 female individuals and 30 male individuals randomly selected from the “Z0” population.

### Generation and Analysis of BSA Sequencing Data

An Illumina HiSeq X Ten platform (Illumina, San Diego, CA, United States) was used for paired-end sequencing of the two bulks and the two parents. The raw sequence data have been submitted to the NCBI’s Sequence Read Archive (SRA) under the BioProject accession number PRJNA719652. Raw reads were processed to get high-quality clean reads according to the following filtering standards: (1) removing reads with the number of N bases accounting for over 10%; (2) removing reads with the number of low-quality bases (phred quality value under 20) accounting for over 50%; and (3) removing adaptor-polluted reads (reads containing more than five adaptor-polluted bases). The Burrows-Wheeler Aligner software program (Li and Durbin, 2009) was used to align the clean reads of each sample against the *Vitis vinifera* grapevine reference genome (12× version). InDel and SNP calling was performed using the Variant Filtration parameter in the Genome Analysis Toolkit (McKenna et al., 2010) with the following settings: cluster Window Size 4, filter “QD < 4.0 || FS > 60.0 || MQ < 40.0”, G filter “GQ < 20”. The ANNOVAR Software Tool (Philadelphia, PA, United States) was used to align and annotate InDels and SNPs and to determine their physical locations in the genome (Wang et al., 2010).

### Mapping of Sex Locus and Candidate Gene Selection

Initial location of sex locus was done by BSA + NGS approach. SNP index was calculated for all variants. The SNP index was the ratio of the number of reads harboring a mutant SNP to that of reads corresponding to the SNP. When all the reads are identical to the reference genome, the index equals 0, whereas when all the reads are different from the reference genome, the index equals 1 (Takagi et al., 2013). ΔSNP index was determined by subtracting the SNP index of F-bulk by that of M-bulk. To reduce the impact of sequencing and alignment errors, we filtered out those variants for which the SNP index was less than 0.3 and the read depth was less than 7 in both bulks. In this study, the crossing parents are perennial woody grapevine, and are highly heterozygous. When BSA sequencing analysis is performed in heterozygous crops, there will be an effect referring to as the “pseudoexchange effect” where random site changes in the reference sequence may result in the opposite notation of the ΔSNP index of the adjacent site (Xue et al., 2017). However, the good positioning ability of |Δ(SNP index)| and its better suitability than Δ(SNP index) in locating trait loci in heterozygous crops had been proved (Xue et al., 2017). Therefore, the |Δ(SNP index)| was used to identify the sex locus in this study. The sliding window analysis was performed to present the SNP index. We used a window size of 2 Mb and a step size of 100 kb as the default settings. |Δ(SNP index)| was obtained for each read depth, with both 95% and 99% confidence interval in 1000 bootstrap replicates (Takagi et al., 2013). The threshold was set at a 99% confidence level to identify candidate region of sex.

Based on the initial candidate region obtained from BSA analysis, the 117 individuals of “Z0” population were all used to fine map the sex locus. InDels and SNPs were uniformly selected within the candidate region. Primers were designed to genotype the two parents first, and markers that were homozygous for the female parent and heterozygous for the male parent were then selected to genotype the “Z0” population. After discarding the InDel markers that amplified ambiguous or no electrophoretic bands and the SNP markers that generated a number of missing data in the mapping population, we finally used the genotypic data of the remaining nine markers, including sex, to perform linkage analysis. The InDel primers were designed using the software Primer 3 (Untergasser et al., 2012). Polymerase chain reactions (PCR) were carried out in a reaction volume of 20 µl, containing 20 ng of DNA, 1 × Taq polymerase buffer (Mg^2+^ plus), 0.15 mmol/L of each dNTPs, 5 pmol of each primer, and 1 U Taq DNA polymerase, with the following amplification protocol: 4 min at 94°C followed by 25 cycles of 1 min at 94°C, 1 min at 60°C, and 1 min at 72°C followed by a final extension of 7 min at 72°C. Amplification products were separated using 10% non-denaturing polyacrylamide gel electrophoresis (PAGE). SNP marker development and genotyping was done using Kompetitive Allele Specific PCR (KASP) technology (Semagn et al., 2014). The trait “*sex*” was scored as a male segregating marker for the paternal parent “043”. The genotypic data were then collected and organized according to the instruction of the software Joinmap 4.0 to perform the linkage analysis (Van Ooijen, 2006). A logarithm of odds score (LOD) of 4.0 was used for grouping, and the genetic distances were calculated using the Kosambi function.

### Cloning of *VaAPRT3*, Sex Marker Development, and Verification

**FIGURE 1:**
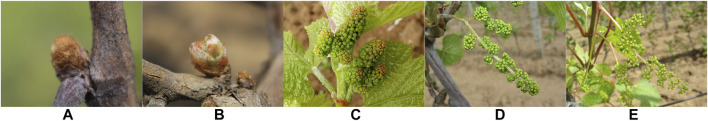
Five developmental stages of buds or flowers of male *V. amurensis*. Females and hermaphrodites (not shown) resemble males in these developmental stages. **(A)** Wooly bud. **(B)** Green tissue visible. **(C)** Flower buds aggregated. **(D)** Aggregated flower buds in separated branches. **(E)** Flower buds separated; ready to bloom.

*VaAPRT3* gene was cloned from both genomic DNAs (gDNA) and complementary DNAs (cDNA) of “Zuoshanyi”, “043”, “75042”, and “Shuangqing”. The gene sequence was firstly downloaded from Genonscope 8× genome version (https://www.genoscope.cns.fr/cgi-bin/ggb/vitis/geneView?src=vitis&name=GSVIVT00007310001). The full length of the DNA sequence of the *VaAPRT3* gene was amplified using Max Master Mix DNA polymerase (Vazyme, Nanjing, China) with the following primer pair: forward, 5′-CTT​TCC​CTC​TCT​CTT​ATC​GA-3′, and reverse, 5′-GGC​CAG​AGG​AAT​TCA​TAC​T-3’. The cDNA from small inflorescence (stage C in Figure 1) was amplified with the following primer pair: forward, 5′-ATG​TCG​GCT​TGC​AAA​GAC-3′, and reverse, 5′-TCA​ATG​GTA​CTC​AAC​TAG​TAT​G-3’. The PCR products with a 3′-A addition were inserted into pEASY-T1 Cloning Vector (TransGen, Beijing, China) using TA-cloning strategy followed by transformation of chemically competent *E. coli* Trans5α cells (Hanahan, 1983). To confirm the genotypes and the transcribed alleles of the three sexes, ten putative clones for DNA and cDNA were sequenced by Sangon Biotech Co., Ltd. (Shanghai, China). Sequence alignment was done using DNAMAN 6 software. According to the polymorphism in the fifth intron of the gene, a primer pair (forward primer: 5′-AGA​AAC​TAG​AGC​CTC​CGG​AA-3’; reverse primer: 5′-AAA​ATC​AAA​GCA​TGG​CCC​CT-3′) was designed to amplify a fragment covering a 22-bp InDel among three sexes. This primer pair was used to amplify the DNA samples of “Zuoshanyi”, “043”, “75042”, and “Shuangqing”, the three cross populations, as well as the small group of *V. amurensis* germplasms listed in Supplementary Table S1.

### RNA Extraction and Expression Analysis of *VaAPRT3*

Using Baggiolini’s description as a reference (Baggiolini, 1952), flowers or flower buds at five developmental stages were collected from “Zuoshanyi”, “043”, and “Shuangqing” (Figure 1). Total RNA of each sample was isolated using the Plant Total RNA Isolation Kit (Sangon Biotech, Shanghai, China) according to the manufacturer’s instructions. cDNA was synthesized using a TransScript® One-Step gDNA Removal and cDNA Synthesis SuperMix (Transgen, Beijing, China) according to the manufacturer’s instructions. A primer pair (forward primer: 5′-ATA​ACG​ACT​CTG​CTA​CTT-3’; reverse primer: 5′-TCC​CTT​CCA​TAT​TCC​AAA-3′) were used for quantitative reverse transcription polymerase chain reaction (qRT-PCR). qRT-PCR was performed in a 20-μl reaction volume containing 10 μl of master mix (ChamQ Universal SYBR qPCR Master Mix, Vazyme, China), 1.6 μl of each primer, and 1 μl of cDNA with the ABI QuantStudio 6 Flex System (Applied Biosystems, Foster City, CA, United States). The relative expression levels of the *VaAPRT3* gene, normalized to grapevine β-actin (forward primer: 5′-CTT​GCA​TCC​CTC​AGC​ACC​TT-3’; reverse primer: 5′-TCC​TGT​GGA​CAA​TGG​ATG​GA-3′), were calculated using the 2^−ΔΔCt^ method with three biological replicates. Standard PCR was performed for semi-quantitative analysis, with 5-min denaturation at 95°C followed by 30 cycles of 95°C for 30 s, 52°C for 30 s, and 72°C for 30 s. The PCR products were analyzed on a 1% agarose gel electrophoresis.

### Measurement of Endogenous Cytokinin Content

Plant samples were the same as described in 2.6. The measurement of endogenous cytokinins was performed as described with minor modifications (Dobrev and Vankova, 2012). In brief, 50 mg of each fresh plant material was frozen in liquid nitrogen, ground into powder, and extracted with methanol/water/formic acid (15:4:1, V/V/V). The combined extracts were evaporated to dryness under nitrogen gas stream, reconstituted in 80% methanol (V/V), and filtered (PTFE, 0.22 μm; Anpel). The extracts were analyzed using an UPLC-ESI-MS/MS system (UPLC, ExionLC™ AD; MS, Applied Biosystems 6500 Triple). The experiments were relegated to Wuhan Metware Biotechnology Co., Ltd. (Wuhan). Three replicates of each assay were performed under the same conditions.

## Results

### Sex Inheritance in *V. amurensis*

Sexual phenotype segregated in all three cross populations used in this study. The segregation of sex fit the single major gene model proposed by Antcliff (1980). Among the 117 individuals of the “Z0” population, 56 and 61 were female and male, respectively. For the “Z7” population, 60 individuals exhibited a female phenotype, and 66 were male. For the “ZS” population, 45 individuals were female and 48 were hermaphroditic. All populations exhibited a 1:1 segregation ratio at a 0.05 level of probability, with the *χ* ^2^ value of 0.14, 0.21, and 0.03 for “Z0”, “Z7”, and “ZS” population, respectively, being less than *χ* ^2^_0.05,1_ = 3.84. It can be concluded from the segregation pattern of the three populations that the sex genotypes for all the female, male, and hermaphroditic individuals, including the four cross parents, were FF, MF, and HF respectively.

### Analysis of BSA Sequencing Data and Mapping of Sex Locus

The Illumina HiSeq X Ten platform generated nearly 49.4 Gb of raw sequencing data (SRA accession: PRJNA719652). A total of 55,848,330, 66,428,654, 100,942,254, and 106,175,722 raw reads were obtained from “Zuoshanyi”, “043”, and the F- and M-bulk, respectively. After removing low-quality, adaptor polluted, and N reads, nearly 312 million of clean reads were produced and 81.63%–89.10% clean reads were mapped on the *V. vinifera* reference genome. The average sequencing depths ranged from 17.78 to 33.80 (Table 1).

**TABLE 1:** Statistics of sequencing data.

Sample	“Zuoshanyi”	“043”	F-bulk	M-bulk
Raw reads	55848330	66428654	100942254	106175722
Clean reads	53015794	61611394	95664044	101763424
Clean reads rate (%)	94.93	92.75	94.77	95.84
Low-quality reads	2317102	4102106	2768868	3424110
Low-quality reads rate (%)	4.15	6.18	2.74	3.22
N reads	536	4035	118	8773
N reads rate (%)	0	0.01	0	0.02
Adaptor polluted reads	514348	707082	2509100	970638
Adaptor polluted reads rate (%)	0.92	1.06	2.49	0.91
Mapped reads	47238906	50291512	85192613	87978214
Mapping rate (%)	89.1	81.63	89.05	86.45
Average depth (X)	17.78	21.15	32.14	33.8

**FIGURE 2:**
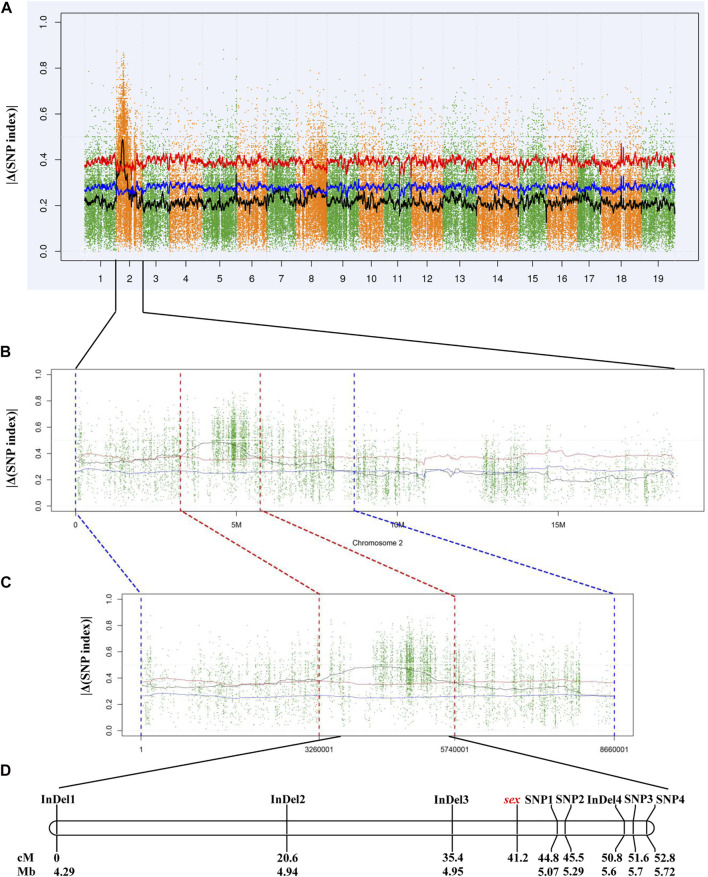
Mapping of sex locus by NGS-based BSA and linkage analysis. **(A)** Genome-wide |Δ(SNP index)| graph. (**B)** |Δ(SNP index)| graph of chromosome 2. (**C)** |Δ(SNP index)| graph in the candidate region. (**D)** Genetic and physical map of the region harboring sex locus. The blue and red lines indicate the confidence level of 0.05 and 0.01, respectively.

In total, 5,403,071 variants, including 4,847,762 SNPs and 555,309 InDels, were detected and to calculate |Δ(SNP index)|. Initial mapping generated a peak of variants in a specific region on chromosome 2 (Figure 2A). According to the |Δ(SNP index)| value, a 2.48-Mb candidate region between 3.26 and 5.74 Mb was identified at a 0.01 confidence level (Figures 2B,C). There were 26,078 SNPs and 3,927 InDels within this 2.48-Mb candidate region (Supplementary Table S2). Then, eight pairs of primers were designed to genotype the 117 individuals of the “Z0” population and to perform linkage analysis (Supplementary Table S3). As a result, a linkage group with a total length of 52.8 cM was constructed. The sex locus was mapped to a 9.4 cM region corresponding to a physical distance of approximately 130 kb on chromosome 2 in the *V. vinifera* reference sequence (Figure 2D).

### Cloning of *VaAPRT3* Gene and Sex Marker Development

**FIGURE 3:**
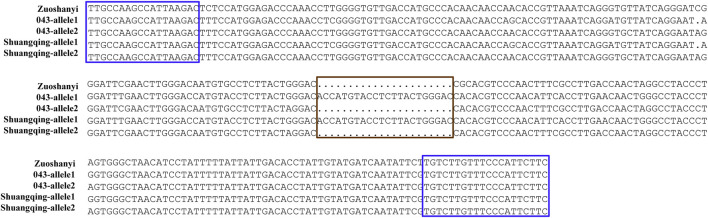
Sequence harboring the 22-bp InDel among three sexes. Brown box: region where male and hermaphroditic individuals have another 22 more bp than female individuals. Blue box: primers used for amplification. This 22-bp InDel corresponds to an insertion between 5195334 and 5195335 bp in chromosome 2 of Gaze annotation of the 8× *V. vinifera* reference sequence version. The location of *APRT3* gene can be indicated in the 8× *V. vinifera* reference sequence version, because it is absent from the 12× version.

**FIGURE 4:**
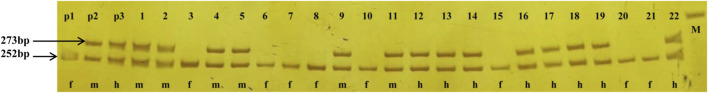
PCR amplification of the sex marker in “Zuoshanyi”, “043”, “Shuangqing” and some individuals of the “Z0” and “ZS” populations. **p1:** “Zuoshanyi”, **p2:** “043”, **p3:** “Shuangqing”, **1–11:** 11 individuals randomly chosen from the “Z0” population, **12–22:** 11 individuals randomly chosen from the “ZS” population, **f:** female individual, **m:** male individual, **h:** hermaphroditic individual, **M:** 300 bp marker.

Based on the *V. vinifera* reference sequence (12× version) and the position estimation of two genes that were absent from the 12× reference sequence version (Fechter et al., 2012) but existed in the 8× version (Picq et al., 2014), a total of 10 genes were located in our 130-kb predicted region (Table 2). Among the 10 predicted genes, we focused on *APRT3* gene. Then, cloning and sequencing results from DNAs showed that the entire gene was homozygous in “Zuoshanyi” but heterozygous in “043”, “75042”, and “Shuangqing”, resulting in dozens of polymorphic sites among the three sexes. A 22-bp insertion in each of the “043”, “75042”, and “Shuangqing”alleles allowed us to develop a potential marker for sex identity (Figure 3). For the female individuals lacking the 22-bp insertion, a single PCR fragment was expected, but for the male and hermaphroditic individuals, another larger fragment was also expected. PCR results confirmed the expected fragment length polymorphism in the “Z0” and “ZS” populations. All female individuals (FF) showed a single band of 252 bp, and all males (MF) and hermaphrodites (HF) displayed both the 252-bp band and a longer 273-bp band (Figure 4). The universality of the marker was then verified in the “Z7” population and a small group of *V. amurensis* germplasms (Supplementary Table S1), and the result was the same with that of the “Z0” population, demonstrating a complete co-segregation relationship between the marker and the trait “*sex*” within *V. amurensis*.

**TABLE 2:** Genes located in the predicted region on chromosome 2 of the 12× reference sequence.


Gene ID	Position	Annotation
GSVIVT01001280001	4951821–4956004	Flavin-containing monooxygenase
GSVIVT01001281001	4957741–4960852	Flavin-containing monooxygenase
GSVIVT01001282001	4962612–4965728	Flavin-containing monooxygenase
GSVIVT01001284001	4974657–4978139	Flavin-containing monooxygenase
GSVIVT01001285001	4983356–4986675	Unnamed protein
GSVIVT01001286001	4989461–4989778	WRKY transcription factor 21
GSVIVT00007310001^a^	5009498–5010308^b^	Adenine phosphoribosyltransferase
GSVIVT01001270001	5025234–5026199	Unnamed protein
GSVIVT00007320001^a^	5036698–5037504^b^	Phosphatidic acid phosphatase 2
GSVIVT01001290001	5062531–5074048	mRNA-oxysterol-binding protein-related protein 2A

a Predicted by the Gaze annotation of the 8× reference sequence.

b Position estimated by Picq et al. (2014).

**FIGURE 5:**
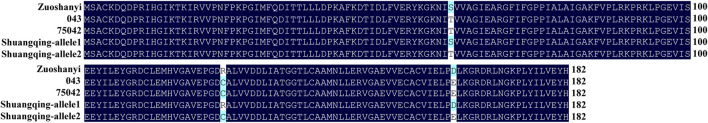
Protein sequence alignment of *VaAPRT3* gene in “Zuoshanyi”, “043”, “75042”, and “Shuangqing”.

Cloning and sequencing of cDNAs from small inflorescences (Stage C in Figure 1) showed that the full length of the open reading frame (ORF) of the *VaAPRT3* gene was 549 bp in all three sexes, potentially encoding a protein of 182 aa. However, “Zuoshanyi” only expressed one allele (named female allele), and each of “043” and “75042” only expressed another allele (named male allele), though the DNA sequence of the *VaAPRT3* gene in “043” and “75042” was heterozygous. The hermaphroditic “Shuangqing” expressed both the female and the male alleles at this stage. Sequence alignment displayed several SNPs among these alleles, and three of them were non-synonymous, causing amino acid changes at position 61, 124, and 163 in the protein sequences (Figure 5). Notably, two of them were located within conserved sites of the *APRT* enzyme, potentially influencing its affinity to different cytokinin substrates.

### Expression Pattern of *VaAPRT3* Gene

**FIGURE 6:**
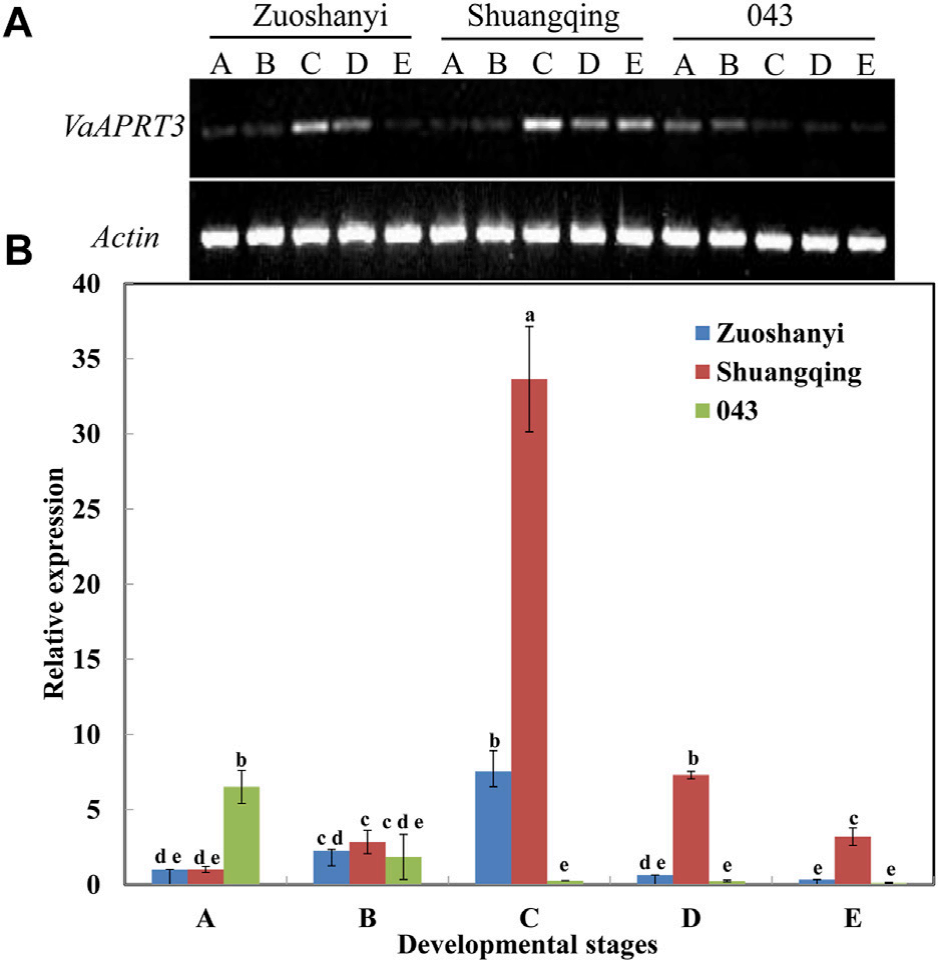
Expression of *VaAPRT3* gene. **(A)** Semi-quantitative PCR results, **(B)** qRT-PCR results. Capital letters A to E correspond to the five developmental stages in Figure 1. Lowercase letters are a marker for the significant differences at the 0.05 level among three sexes at each single stage.

To examine the expression characteristics of the *VaAPRT3* gene in three different sexes, we selected the female “Zuoshanyi”, one male “043”, and the hermaphroditic “Shuangqing” to perform semi-quantitative PCR and qRT-PCR analysis. The results showed that for female “Zuoshanyi” and hermaphroditic “Shuangqing”, the expression of the *VaAPRT3* gene displayed an increasing–decreasing pattern during flower development process, and reached the peak at stage C. However, for male “043”, it exhibited a continuous decreasing pattern. Considering each single developmental stage, the relationship of gene differential expression was “Shuangqing” > “Zuoshanyi” > “043” at stage C, D, and E. However, at early stage A, the gene expressed significantly higher in “043” than in “Shuangqing” or “Zuoshanyi” (Figure 6).

### Contents of Cytokinins

**FIGURE 7:**
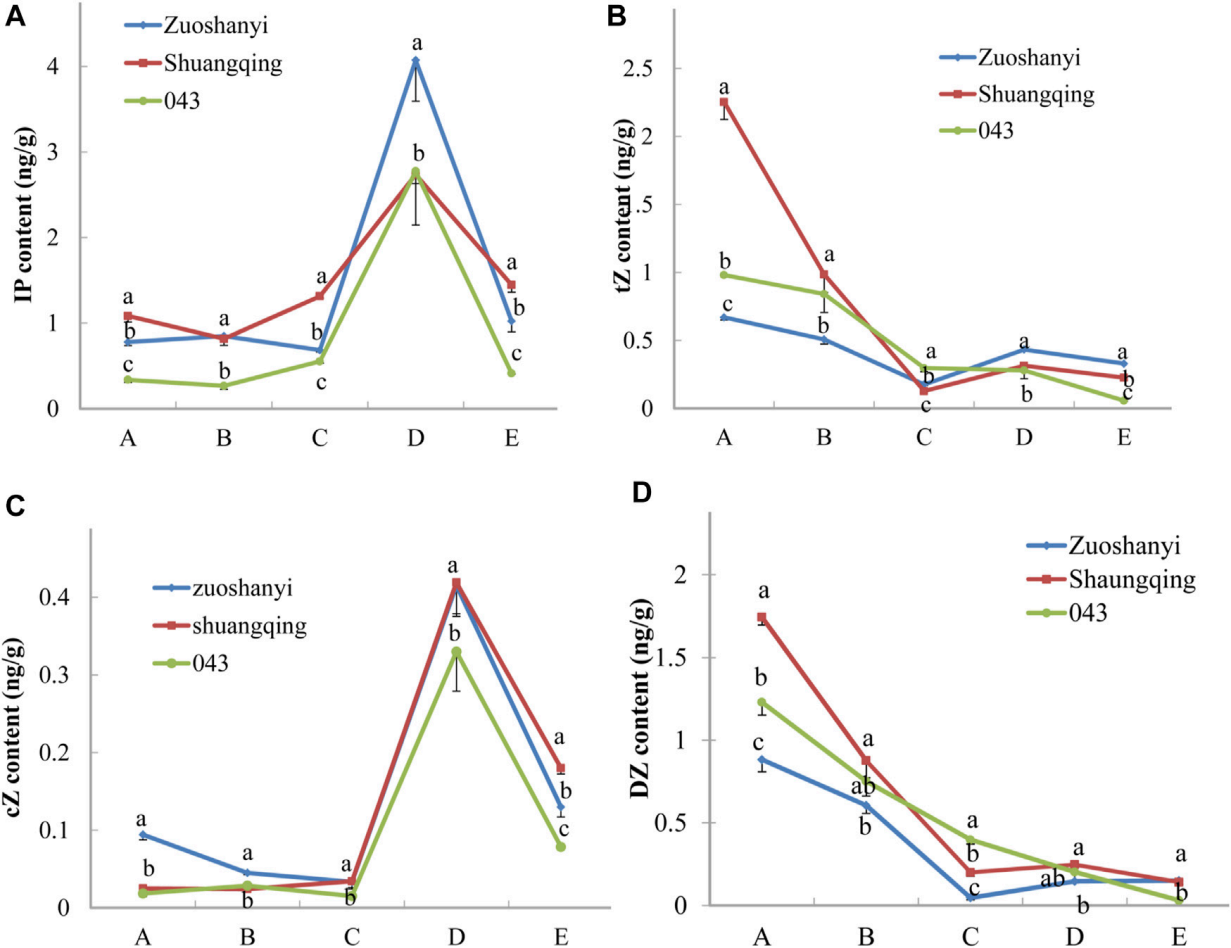
Contents of iP, tZ, cZ, and DZ. Capital letters A to E correspond to the five developmental stages in Figure 1. Lowercase letters are a marker for the significant differences at the 0.05 level among three sexes at each single stage.

To investigate the affinity of certain *VaAPRT3* alleles to cytokinin substrates, we measured the contents of four active cytokinin nucleobases including N6-(Δ2-isopentenyl) adenine (iP), trans-zeatin (tZ), cis-zeatin (cZ), and dihydrozeatin (DZ) in three sexes (Figure 7). The materials used here were the same with 3.4. For tZ and DZ, the content exhibited a continuously decreasing pattern in male “043”; however, in female “Zuoshanyi” and hermaphroditic “Shuangqing”, it touched a lowest point at stage C and then increased at later stage. Therefore, a negative relationship could be found between the expression level of the *VaAPRT3* gene and the contents of tZ and DZ in “Zuoshanyi” and “Shuangqing” at stage C, which we consider as the start point of the unisexual stage of sex differentiation in *V. amurensis*. At stage C, the female allele of the *VaAPRT3* gene was expressed only in female “Zuoshanyi” and hermaphroditic “Shuangqing” whose pistil normally developed, indicating a special affinity of female allele of the *VaAPRT3* gene to tZ and DZ. In our previous study, the development of pistil, which was supposed to abort in male “043” flowers, was recovered under the treatment of N1-(2-chloro-4-pyridyl)-N3-phenylurea (CPPU) at a time point just near stage C described in this present study (Shen et al., 2018). So, taking all the information together, we consider stage C as the key time point of sex differentiation, or more accurately, pistil differentiation. However, for iP and cZ, the content changing trends were almost the same in three sexes, especially after stage C, so we excluded their correlation with sex differentiation. We hypothesized that an internal environment of low tZ or DZ content at a certain stage was necessary for the initiation of pistil development, and this is driven by the high expression of female allele of the *VaAPRT3* gene at that certain stage. The later increase of cytokinin (iP and cZ) content might be needed for floral organ development.

## Discussion

### Genetics of Sex in *V. amurensis*

In this study, we confirmed the single sex locus theory and the allelic dominance of M > F and H > F using 3 *V. amurensis* intraspecific cross populations. Although the dominance relationship between M and H cannot be illustrated here due to the lack of an HH × MF or an HF × MF crossing pattern as described by Dalbó et al. (2000) and Marguerit et al. (2009), a previous study in pure *V. amurensis* revealed a 2:1:1 segregating ratio of male, hermaphroditic, and female progenies from the crossing pattern HF × MF, proving the dominance of M over H (Yin et al., 1997). So, altogether, the dominance relationship was M > H > F in *V. amurensis*, being consistent with those in other *Vitis* species (Marguerit et al., 2009; Fechter et al., 2012; Battilana et al., 2013; Hyma et al., 2015).

### The Genetic and Physical Distance of the Linkage Group

In our study, the total genetic distance of the linkage group was 52.8 cM, and the linkage group was constructed based on molecular markers within a 1.43-Mb physical distance, whereas in the *V. vinifera*-derived reference genetic linkage maps, the average distance of a single chromosome was no more than 86.4 cM (Adam-blondon et al., 2004; Riaz et al., 2004), and the total physical length of chromosome 2 in the *V. vinifera* grapevine reference genome (12× version) was 18.78 Mb. So, in our linkage group, comparatively, the small physical distance corresponded to a very large genetic distance. What is more, there exists discrepancy in the ratio of physical to genetic distance in our linkage group. For example, between the two adjacent markers InDel1 and InDel2, the ratio was 31.6 kb/cM, but the ratio was only 0.68 kb/cM between InDel2 and InDel3. This phenomenon was also observed in other plants, such as barley, where long genetic distances translated into short physical distances, and *vice versa*, and that was thought to be related to different frequencies of recombination events at different chromosome regions (Bustamante et al., 2017). However, based on our results, it is hard to clarify whether there exists unequal distribution of recombination frequency along the linkage group, because the limitation of mapping population size and marker number used for map construction may influence the uniformity of marker distribution, thus affecting the ratio of physical to genetic distance. Although the linkage group in our study is hardly to be of high quality, the mapping of sex locus is satisfactory, being consistent with previous published sex locus in other *Vitis* species (Badouin et al., 2020; Fechter et al., 2012; Picq et al., 2014; Lewter et al., 2019; Massonnet et al., 2020).

### Sex Locus and Candidate Sex Genes in *Vitis*

The sex locus defined in *Vitis* species almost corresponded to a same small region in the genome, including a 143-kb region in *V. riparia* or *V. cinerea* (Fechter et al., 2012), a 151.8-kb region in *V. v. sylvestris* (Picq et al., 2014), a 111-kb region in *V. v. sylvestris* (Badouin et al., 2020), a 257-kb region in the Cabernet Sauvignon genome (Massonnet et al., 2020), the 130-kb region in *V. amurensis* by us, and even a 450-kb region in the subgenus *Muscadinia* (Lewter et al., 2019). The colocalization of the sex locus across different *Vitis* species could be explained by the estimated age of the sex-determining region (Charlesworth, 2013). In grapevine, the sex locus could be 112.5 million years old (Badouin et al., 2020). However, the separation of the *Vitis* and *Muscadinia* subgenera was only thought to have occurred 18 million years ago (Wan et al., 2013). That means the sex locus was formed much earlier than the divergence of *Vitis* species. So, the formation of the small sex region can be a very ancient event in grapevine. The small size of the region could result from rare recombination between M and F haplotypes, preventing its evolution to a full chromosome in *Vitis* (Picq et al., 2014). Although small in size, the region contains several genes proved to have a potential impact on sex determination. An *APRT3* gene, based on which a sex marker was developed to distinguish females from males/hermaphrodites, might be a candidate contributing to sex determination in *Vitis* (Fechter et al., 2012). A *VviFSEX* gene was considered as a candidate responsible for male sterility, while a *VviAPRT3* gene was proved functional in the arrest of carpel development (Coito et al., 2017). They allowed the distinction of three different sex phenotypes among *V. vinifera* individuals when used simultaneously (Coito et al., 2017). In a recent comprehensive study regarding grapevine sex genetics, a recessive allele of *VviINP1* gene containing an 8-bp deletion interrupted male function, making *VviINP1* a plausible male-sterility candidate, and another M-linked transcription factor *VviYABBY3* was postulated to be associated with female sterility (Massonnet et al., 2020). In this study, the *VaAPRT3* gene was hypothesized to be associated with female organ initiation in *V. amurensis.* In all, multiple genes may be involved in different sexual organ formation in grapevine, illustrating a complexity of sex determination mechanism in *Vitis*.

### Role of *APRT* Gene in Sex Determination

In our study, the candidate sex-determining gene *VaAPRT3* was highly expressed in female and hermaphroditic flowers at the key developmental stage (stage C in Figure 6), but in a previous study regarding sex specification in *V. vinifera* subs *sylvestris*, the *VviAPRT3* gene showed a significant high expression in male flowers compared with female or hermaphroditic flowers at a similar developmental stage (Coito et al., 2017). These two opposite results both explained the role of the *APRT3* gene in female organ development. Interestingly, in *Arabidopsis thaliana*, the APRT-deficient mutants have been reported to be male sterile due to the failure of microspore mitotic division (Moffatt and Somerville, 1988; Regan and Moffatt, 1990). So based on these results, different homologues of *APRT* gene may play different roles in different plant species, or they have different function mechanisms though for the same genus but different species.

The sex determination mechanism hypothesized in this study was based on the combination of differential expressions of the *VaAPRT3* gene and endogenous cytokinin contents between different sexes. As for the function of the amino acid changes in the protein sequence of the *VaAPRT3* gene, we do not have experimental evidence to prove their role in sex determination; thus, this is worth researching in the next step. Recently, an interesting result was obtained by a phylogenic analysis tending to divide the sex-determining region of grapevine into two parts, where the first 8-kb part was M-linked potentially responsible for female sterility, and the F-linked part started from 40 kb downstream, suggesting a possible role in male sterility (Massonnet et al., 2020). This may imply that the development of male and female in grapevine was controlled by two independent systems, and can also explain why the *VaAPRT3* gene identified in this study is only available to explain female organ differentiation. In the future, with the help of recently published genome of *V. amurensis* (Wang et al., 2021), more information will be available for studies on this species. It is also necessary to investigate other genes in the candidate sex region of *V. amurensis*, and to uncover the sex determination mechanism comprehensively through various experimental methods, such as gene editing and transgenic verification (Akagi et al., 2014; Akagi et al., 2018; Akagi et al., 2019).

### The Sex Marker for *V. amurensis*

The perennial woody grapevine has a large plant size and a long juvenile period, which prolong the breeding process. Molecular markers, especially the ones co-segregating with the target trait, can be very useful for selecting desired individuals at the young seedling stage, thus reducing the cost of time and labor as well as economic input. Chinese researchers developed molecular markers related to sex in *V. amurensis* (Tang et al., 2008), but the marker was neither co-segregated with sex nor tested in any cross populations. Here, we developed a sex marker in *V. amurensis* depending on the polymorphism inside the candidate gene *VaAPRT3* among different sexes. No recombinant individual was found in any of the three cross populations or the natural *V. amurensis* germplasms used in this study, suggesting the co-segregant character of the marker, at least based on the population size of this study. This marker could be further used in *V. amurensis* breeding programs, to remove fruitless male individuals in an FF × MF cross population and to retain self-fruitful ones in FF × HF or HF × HF cross populations. As the allelic bands of M and H are identical, the marker can also help distinguish hermaphrodites from males in an HH × MF cross population. The marker can also facilitate the work of investigation and collection of *V. amurensis* germplasm resources, which dates back to the middle of the 19th century (Li et al., 2015). The investigation and collection work, to a great extent, lead to the establishment of the National Field Gene Bank for Amur Grapevine funded by the Chinese Ministry of Agriculture. However, only a small number of male individuals were collected and conserved, and that may be caused by the lopping of male plants by settlers near the mountains, who lacked the awareness of resource conservation. That is also the reason why only a few natural male individuals were used in this study. In the future, to make full use of *V. amurensis* resources and to keep sex balance of this precious species, the work of investigation and collection is still needed. The marker developed in this study will be very helpful in sex identification when the germplasm resources were at the plantlet stage.

## Conclusion

In *Vitis amurensis*, we developed a molecular marker for sex identification based on a 22-bp InDel within the sex candidate gene *VaAPRT3*. This marker is able to distinguish female individuals from males or hermaphrodites in *V. amurensis*. By analyzing gene polymorphism and expression level as well as endogenous cytokinin content, we hypothesize that the *VaAPRT3* gene was involved in sex determination through regulating cytokinin metabolism in *V. amurensis*. Our findings enriched the theory of sex determination in the genus *Vitis*.

## Data Availability

The datasets presented in this study can be found in online repositories. The names of the repository/repositories and accession number(s) can be found in the article/Supplementary Material.
